# Genetic diversity of Saudi native chicken breeds segregating for naked neck and frizzle genes using microsatellite markers

**DOI:** 10.5713/ajas.18.0041

**Published:** 2018-05-31

**Authors:** Moataz Fathi, Mohamed El-Zarei, Ibrahim Al-Homidan, Osama Abou-Emera

**Affiliations:** 1 Department of Animal Production and Breeding, College of Agriculture and Veterinary Medicine, Qassim University, Al-Qassim 51452, Saudi Arabia; 2 Department of Poultry Production, Faculty of Agriculture, Ain Shams University, Hadayek Shoubra 11241, Cairo, Egypt; 3 Department of Animal Production, Faculty of Agriculture, Suez Canal University 41522, Ismailia, Egypt; 4 Department of Poultry Breeding, Animal Production Research Institute, Agriculture Research Center, Dokki, Giza 12618, Egypt

**Keywords:** Genetic Diversity, Microsatellites, Saudi Native Chicken

## Abstract

**Objective:**

Recently, there has been an increasing interest in conservation of native genetic resources of chicken on a worldwide basis. Most of the native chicken breeds are threatened by extinction or crossing with ecotypes.

**Methods:**

Six Saudi native chicken breeds including black naked neck, brown frizzled, black, black barred, brown and gray were used in the current study. The aim of the current study was to evaluate genetic diversity, relationship and population structure of Saudi native chicken breeds based on 20 microsatellite markers.

**Results:**

A total of 172 alleles were detected in Saudi native chicken breeds across all 20 microsatellite loci. The mean number of alleles per breed ranged from 4.35 in gray breed to 5.45 in normally feathered black with an average of 8.6 alleles. All breeds were characterized by a high degree of genetic diversity, with the lowest heterozygosity found in the brown breed (72%) and the greatest in the frizzled and black barred populations (78%). Higher estimate of expected heterozygosity (0.68) was found in both black breeds (normal and naked neck) compared to the other chicken populations. All studied breeds showed no inbreeding within breed (negative inbreeding coefficient [F_IS_]). The phylogenetic relationships of chickens were examined using neighbor-joining trees constructed at the level of breeds and individual samples. The neighbor-joining tree constructed at breed level revealed three main clusters, with naked neck and gray breeds in one cluster, and brown and frizzled in the second cluster leaving black barred in a separate one.

**Conclusion:**

It could be concluded that the genetic information derived from the current study can be used as a guide for genetic improvement and conservation in further breeding programs. Our findings indicate that the Saudi native chicken populations have a rich genetic diversity and show a high polymorphism.

## INTRODUCTION

The importance of keeping genetic diversity in animals and poultry is supported worldwide by the Food and Agriculture Organization [1]. Most of native chicken are threatened by extinction or crossing with ecotypes. However, there has been a greater attention in recent years to the loss of biodiversity and extinction of poultry breeds [2]. It is well known that native chickens have socio-cultural and economic importance in the livelihoods of rural sector and households. There are many native chicken breeds segregating for major genes in Arabian Peninsula that are adapted for high environmental temperature and unfavorable conditions. Saudi consumers prefer native chickens and their products for meat and eggs. Moreover, as a consequence of natural selection, indigenous breeds have shown to possess superior disease resistance [3,4]. Fanciers believe that the naked neck and frizzle genes were brought into Saudi Arabia from European ecotype chicken strains in the ninth century.

It can be assumed that indigenous breeds contain genes and alleles relevant to their adaptation to particular environmental circumstances and specific breeding strategies [5,6]. Using microsatellite markers as a tool for genetic diversity analysis among chicken breeds are well established [7–10]. It is an effective method to assess genetic diversity within and between chicken populations because they are highly polymorphic, show co-dominant inheritance, found to be abundant and evenly distributed throughout the genome [11–14]. Recently, few studies of genetic polymorphism were initiated on Saudi native chicken populations using mtDNA [15,16] or microsatellite markers [4]. Due to the limited mtDNA diversity, it is necessary to analyze autosomal markers in Saudi native breeds, in order to study diversity and the relationship between breeds.

To our knowledge, Saudi native breeds segregating for major genes such as naked neck and frizzle genes were not included in any study. Therefore, the current study aimed at investigating the genetic diversity, relationship and population structure of Saudi native chicken carrying naked neck and frizzle genes based on 20 microsatellite markers.

## MATERIALS AND METHODS

### Native chicken populations

**Figure 1:**
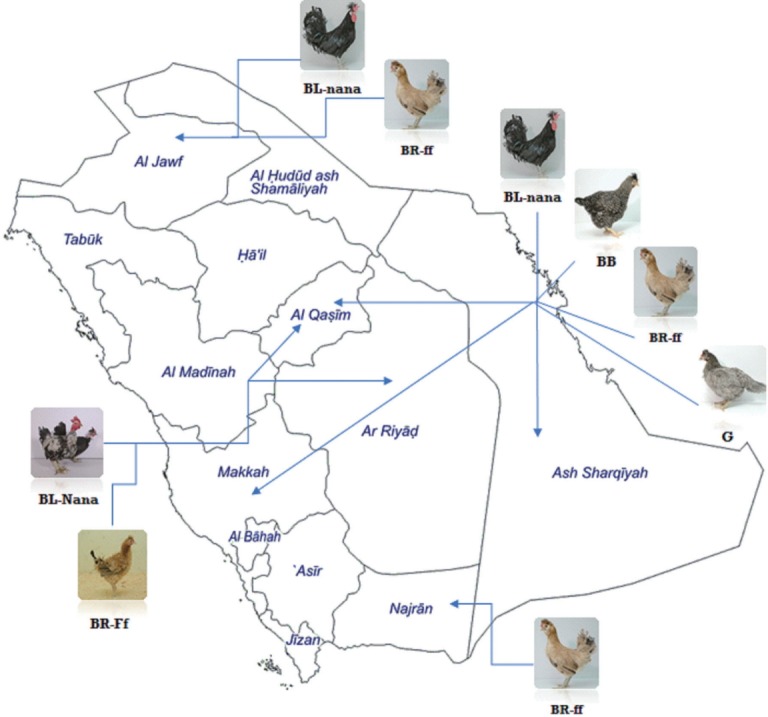
Geographical location of Saudi native chicken populations.

A total of 2,700 one-day old Saudi native chicks, representing six breeds (naked neck black, frizzled brown, black, black barred, brown and gray) (450 each) were kept in the poultry research station, Qassim University. The present breeds were collected and propagated under an extensive breeding program to purify and conserve native genetic recourses. All breeds were kept under similar management and environmental conditions. The care and handling of chickens were in accordance with regulations of animal care and ethics committee of Qassim University. The name, abbreviation, morphological appearance and photo for each population are summarized in Table 1. Also, geographical location of Saudi native chicken populations is shown in Figure 1.

### DNA extraction and microsatellite genotyping

A total of 144 blood samples were collected from the different chicken populations (24 each). Approximately one mL blood/bird from a wing vein was collected in ethylenediamine tetraacetic acid (EDTA) tubes and stored at −20°C. DNA was extracted from 0.5 mL of whole EDTA blood using ILLUSTRA blood mini spin kit (GE Life Sciences, Buckinghamshire, UK). Then, DNA samples were taken to determine the quantity and quality of DNA using Thermo Scientific Nano Drop 8000 UV-Vis Spectrophotometers. The selected microsatellites were amplified using ProFlex PCR System Applied Biosystems (Toronto, ON, Canada). The polymerase chain reaction (PCR) was performed for each locus in 10 μL reactions consisted of 2 μL of Genomic DNA (20 ng), 5 μL 2×PCR AmpliTag gold PCR Master mix (Applied Biosystems, Foster City, CA, USA), 0.4 μL primer mix (50 pmoles) and 2.6 μL DNase free water. The PCR program was carried out at 95°C for 5 min, followed by 30 cycles of 95°C for 30 s. Annealing temperature (ranged from 58°C up to 64°C depending on primers sequence) was determined for each primer for 30 s. and 72°C for 30 s, and final extension at 72°C for 10 min. Following the completion of the PCR cycles, 3 μLof the reaction products was mixed with 1 μL 6×gel loading dye and then loaded into each well of vertical 8% polyacrylamide gel made with 1×tris base, boric acid and EDTA buffer at 100 V for 60 to 90 min and stained with Ethidium bromide (1%). A 50 bp DNA ladder was used to estimate allele sizes in base pairs (bp). A reference bird was used to compare and correct allelic size in each gel. Twenty microsatellite markers distributed on 14 autosomal chromosomes and previously used by Fathi et al [4] and recommended by the International Society of Animal Genetics (ISAG)-FAO [1] were selected to assess the genetic diversity among Saudi native chicken breeds (Table 2).

### Data analysis

Genetic variability was estimated per locus and across all loci for each population by allelic frequencies, observed heterozygosity, expected heterozygosity in all populations (as estimated from the pooled allele frequencies [H_T_]), expected heterozygosity estimated within a population (H_S_) and Hardy Weinberg equilibrium (HWE) using GENETIX program [17] and GENEPOP 4.1 software [18].

The F_IT_ (inbreeding coefficient of an individual relative to the total population), F_ST_ (the effect of subpopulations compared with the total populations), and F_IS_ (inbreeding coefficient of an individual relative to the subpopulation) and values for each breed were calculated using the FSTAT 2.9.3 [19] and GENEPOP 4.1 software [18]. The following formulas were used to calculate their values:

$$\begin{array}{l} {\text{F}}_{\text{IT}}=1-\frac{HI}{HT}\\ {\text{F}}_{\text{IS}}=1-\frac{HI}{HS}\\ {\text{F}}_{\text{ST}}=1-\frac{HS}{HT} \end{array}$$

Where, HT, total gene diversity or expected heterozygosity in the total population as estimated from the pooled allele frequencies; HI, intra-population gene diversity or average observed heterozygosity in a group of populations; HS, average expected heterozygosity estimated from each subpopulation.

Genetic distance was computed between populations without a bias correction [20]. These genetic distances were developed based on different models of molecular evolution and/or different taxonomic units [21]. The POPTREE2 program V. 2.0 [22] was used to perform evolutionary analyses of allele frequency data to construct a phylogenetic tree for the studied breeds. A neighbor-joining (NJ) tree of the seven chicken populations was constructed.

## RESULTS AND DISCUSSION

### Ploymorphism of markers

Across the 20 microsatellite markers studied, a total of 172 alleles were identified in all native chicken breeds (Table 3). All studied loci were polymorphic in all chicken breeds. Overall, the lowest number of alleles per locus (5 alleles) was recorded for MCW0034 and MCW00165, while the highest number (15 alleles) was recorded for LEI0234. The average number of alleles per locus was 8.6. The highest mean number of alleles was recorded in the black breed (5.5), whereas, the lowest number (4.4) was recorded in gray breed. However, FAO has specified a minimum of 4 distinct alleles per locus for efficient judgment of genetic diversity between breeds. Generally, both black chicken genotypes (naked neck and normally feathered) exhibit a high genetic diversity in terms of number of alleles. This may be due to the fact that the black chickens are more popular and are distributed in a wide area. The lowest allelic diversity observed in gray breed may also suggest a high degree of inbreeding. The mean number of alleles of Saudi native populations was higher than that of Taiwanese conserved breeds [23] and Vietnamese domestic chicken populations [24]. Compared with our findings (8.6 alleles), Shahbazi et al [25] reported a mean number of alleles of 4.8 per locus in Iranian native chickens. While, Kaya and Yildiz [26] reported that the mean number of alleles among studied loci for Turkish native chickens was 7.5. These values were lower than those reported by Zhang et al [7], who stated that there were 9.3 alleles in Chinese native chicken breeds. However, the number of alleles at a single microsatellite locus in any single chicken population has ranged from one (monomorphic) up to several [27,28]. The average of alleles per locus observed ranged from 3.4 to 9.3 in non-commercial birds [25,26,29,30]. The higher values of alleles in non-commercial chickens in relation to the commercials are due to a strong pressure of artificial selection which occurred in the latter.

### Fixation indices and heterozygozity

Fixation indices (F_IT_, F_ST_, and F_IS_) estimated according to Weir and Cockerham [31] are listed in Table 4. Also, the estimates of observed heterozygosity (H_O_) and expected heterozygosity (H_E_) were recorded based on allele frequency data for each locus per breed (Table 4). The average of observed heterozygozity was quite high (0.76) with a range of 0.35 to 0.91. The MCW0123 marker recorded the lowest figure (0.35), while the MCW0183 marker recorded the highest one (0.91). Expected heterozygosity recorded an average of 0.71 across all loci, ranging from 0.44 (MCW0123) to 0.83 (MCW0165). Similar average was found (0.71) for total gene diversity (H_T_) across all native chicken breeds. However, the mean of H_E_ recorded in the current study was lower than that reported by Zhang et al [7] in Chinese native chickens. On the other hand, H_E_ was higher than that of Hillel et al [32], who reported that the average gene diversity within 52 breeds across all 22 loci was 0.47. The variation of expected heterozygosity may be due to the differences in location, sample size, breed structure and microsatellite markers [33]. The highest value of total gene diversity (H_T_) for all studied breeds was 0.85 (MCW016), which meant that this locus was a highly informative locus among all loci. On the other hand, the value of H_T_ for MCW012 was 0.48, indicating a slightly informative locus.

The F_IS_ represents a degree of nonrandom mating (deviation from HWE). A positive number for F_IS_ means deviation from HWE. Only MCW0069, MCW0165, MCW0103, MCW0123, and MCW0295 showed a positive number. Out of all used markers, 75% showed negative figures. Relatively, low average F_IS_ (−0.08) across all loci in the resent study was recorded, indicating non-random mating in the Saudi native chicken breeds. However, F_IS_ is used to obtain a deeper insight in appraising the degree of in-breeding and endangerment potentiality and is considered as an important tool to judge the conservation priority [34]. Accordingly, when F_IS_ is less than 0.05, the breeds are not in danger. On average, the genetic differentiation index, F_ST_, among breeds was 0.02 (Table 4). About 2% of the total genetic variation corresponded to the differences between breeds and the remaining 98% was the result of variation among individuals within breed. Ramadan et al [34] found a 8% of genetic differentiation across 21 studied loci used among six Egyptian local strains. A higher estimated value of F_ST_ (0.357) owing to line differences was recorded in pure-bred commercial chicken [35].

The expected heterozygosity was lower than the observed heterozygosity for all chicken breeds (Table 5). All populations were characterized by a high degree of genetic diversity, with the lowest heterozygosity found in the brown breed (72%) and the greatest in the frizzled and black barred populations (78%). Higher estimates of expected heterozygosity (0.68) were found in both black breeds (normal and naked neck) compared to the other chicken populations. Moreover, the normally feathered brown breed recorded the lowest expected heterozygosity (0.62). In comparison with naked neck and frizzled chickens kept mainly by fanciers, normally feathered Saudi native chickens exhibited a lower degree of heterozygosity. However, native chickens carrying major genes are kept by fanciers as ornamental breeds in small size populations in many regions of central and south area of Saudi Arabia. This condition seems to result in a higher degree of diversity than the other breeds. Normally feathered black breed had the highest genetic variability in terms of expected heterozygosity (0.68) and number of alleles (5.45). This may be due to the fact that the black native chickens has been found in large number and distributed in a wide area of Saudi Arabia. BB and BR-Ff breeds recorded the highest difference between observed and expected heterozygosity. The H_O_ and H_E_ observed in Saudi native chicken breeds in the current study were similar to or slightly lower than those reported by Zhang et al [7] in Chinese native breeds.

All studied breeds showed no inbreeding within breed (negative inbreeding coefficient [F_IS_]). The mean F_IS_ for Saudi native chicken breeds was lower than zero (−0.07). These values were similar to that reported by Tadano et al [36] for 12 commercial chicken lines (0.000 to 0.141) based on 40 microsatellite loci, while it was lower than that reported by Kaya and Yildiz [26] and Ding et al [37] for Turkish and Chinese native chicken breeds, respectively. To decrease F_IS_, full- or half-sib mating should be avoided to prevent inbreeding depression. Among breeds, the mean F_IS_ values varied from −0.01 for naked neck black breed to −0.10 in both normally feathered brown and black barred. The mean F_ST_ value of 0.02 indicates that approximately 2% of the total genetic variation is caused by breed differences, whereas the remaining amount (98%)was due to differences among individuals within breeds. With respect to HWE, black chicken breeds (either normal or naked neck) and normal brown breed exhibited a deviation from Hardy–Weinberg equilibrium (dHWE). Out of all 120 HWE tests in 6 breeds tested, dHWE at the 5% level recorded 12.9%. Contrary with our results, Ding et al [37] reported that all breeds showed statistically significant deviation from the HWE at many loci.

### Genetic distance among Saudi native chicken breeds

Molecular information of genetic diversity is playing an important role in conservation of chicken resources. Genetic distance based on Nei’s unbiased values is presented in Table 6. The high genetic diversity in Saudi native chicken breeds is consistent with great phenotypic variation among them. The greatest genetic distance was found between gray and brown breeds (0.246).The smallest figure was recorded between the naked neck and normally feathered black populations (0.107). Both normally feathered and naked neck Black breeds (0.107) were most closely related to each other than that of normally feathered and frizzle brown populations (0.139). According to many reports, local chickens from Africa and Asia are characterized by extensive phenotypic variations within and between different populations [35,38–46]. Similarly, high microsatellite diversity was reported within chicken strains from Iran, China, Turkey, Korea, Sudan, Southeast African countries [25,26,35,47,48]. Uncontrolling breeding programs and traditional management systems may be contribute to higher variations within and between these populations. Consistent with our findings, Osman et al [49] and Tadano et al [36] reported that the use of 20 microsatellite markers and approximately 24 birds per breed might be adequate to obtain accurate results of genetic diversity in Japanese chicken breeds.

The unrooted NJ tree derived from the Nei’s standard genetic distance of six Saudi native chicken breeds is given in Figure 1. It could be observed that three main clusters were clearly recognized. Frizzled brown breed and normally feathered brown breed located in one cluster, while naked neck black breed with black-barred breed located in the second cluster. Finally, normal black breed and the gray breed were located in the third cluster. The black and gray breeds were placed to the cluster whose distance is far from naked neck black and black barred breeds. Additionally, naked neck black breed (BL-Nana) grouped with the black barred one (BB).

## CONCLUSION

In conclusion, determining genetic biodiversity between chicken breeds is considered as an initial step of strategic plan for genetic characterization and conservation of Saudi native chickens. Evaluation of genetic diversity among Saudi native chicken breeds based on 20 microsatellite markers studied in the current study was efficient and gained reliable results.

## Figures and Tables

**Figure 2:**
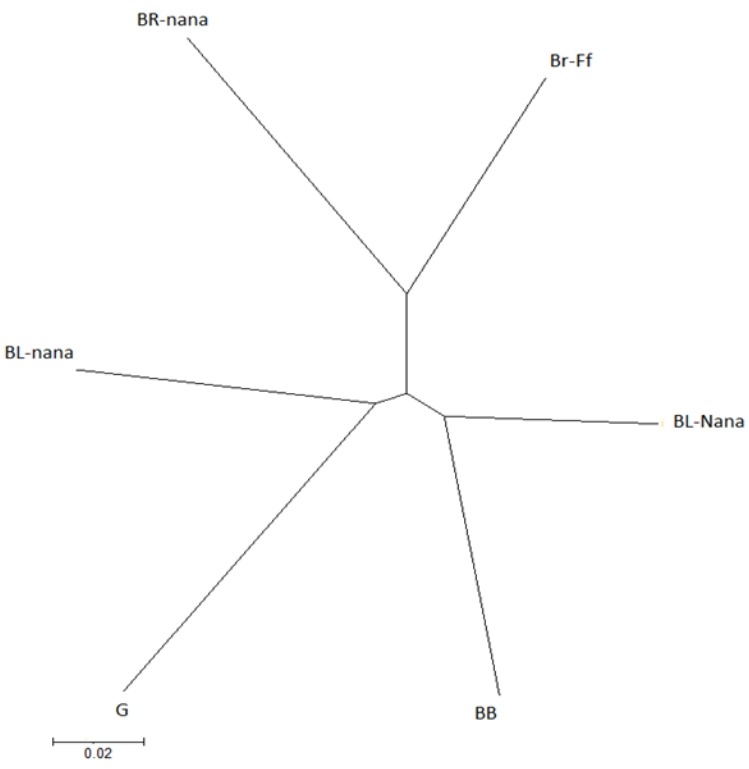
Unrooted neighbor-joining tree constructed using Nei’s genetic distance of Saudi native chicken breeds.

**Table 1:** Description of morphological appearance of the Saudi native chicken populations

Breed	Abbreviation	Morphological appearance/description	Photo
Normally feathered black	BL-nana	The predominant plumage color for males and females is a solid shiny black. Both sexes are entirely black as adults; name derived from plumage color appearance.	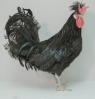
Black barred	BB	The predominant plumage color for both sexes is black with white stripes. The name is derived from plumage pattern.	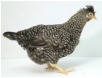
Normally feathered brown	BR-ff	Plumage color ranges from light to dark brown, sometimes with black feathers on the tail. Sometimes feather’s face is outwards.	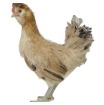
Gray	G	Gray feathers are predominate. Crested head is more frequent than in the other breeds.	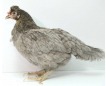
Heterozygous naked neck black	BL-Nana	Multi-colored feather coat with various plumage patterns. Black color is more frequent with medium sized and ornamental appearance. Found in hot middle and southern regions of KSA	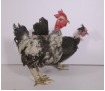
Heterozygous frizzled brown	BR-Ff	The predominant plumage color for both sexes is dark brown with black feathers in abdomen and tail. Carrying various comb types. Found in a village of central region, Al-Qassim province, KSA	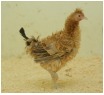

**Table 2:** Description of 20 microsatellite markers used in the current study

No.	Primer	Chromosomal location	Primer sequence (5′→3′), Forward and reverse	Annealing Temp. (°C)	Allele size range (bp)
1	MCW0248	1	GTTGTTCAAAAGAAGATGCATG TTGCATTAACTGGGCACTTTC	60	205–225
2	MCW0111	1	GCTCCATGTGAAGTGGTTTA ATGTCCACTTGTCAATGATG	60	96–120
3	ADL0268	1	CTCCACCCCTCTCAGAACTA CAACTTCCCATCTACCTACT	60	102–116
4	MCW0020	1	TCTTCTTTGACATGAATTGGCA GCAAGGAAGATTTTGTACAAAATC	60	179–185
5	LEI0234	2	ATGCATCAGATTGGTATTCAA CGTGGCTGTGAACAAATATG	60	216–364
6	MCW0206	2	ACATCTAGAATTGACTGTTCAC CTTGACAGTGATGCATTAAATG	60	221–249
7	MCW0034	2	TGCACGCACTTACATACTTAGAGA TGTCCTTCCAATTACATTCATGGG	60	212–246
8	MCW0103	3	AACTGCGTTGAGAGTGAATGC TTTCCTAACTGGATGCTTCTG	64	266–270
9	LEI0166	3	CTCCTGCCCTTAGCTACGCA TATCCCCTGGCTGGGAGTTT	60	354–370
10	MCW0295	4	ATCACTACAGAACACCCTCTC TATGTATGCACGCAGATATCC	60	88–106
11	MCW0081	5	GTTGCTGAGAGCCTGGTGCAG CCTGTATGTGGAATTACTTCTC	60	112–135
12	MCW0014	6	TATTGGCTCTAGGAACTGTC GAAATGAAGGTAAGACTAGC	58	164–182
13	MCW0183	7	ATCCCAGTGTCGAGTATCCGA TGAGATTTACTGGAGCCTGCC	58	296–326
14	ADL0278	8	CCAGCAGTCTACCTTCCTAT TGTCATCCAAGAACAGTGTG	60	114–126
15	MCW0067	10	GCACTACTGTGTGCTGCAGTTT GAGATGTAGTTGCCACATTCCGAC	60	176–186
16	MCW0104	13	TAGCACAACTCAAGCTGTGAG AGACTTGCACAGCTGTGTACC	60	190–234
17	MCW0123	14	CCACTAGAAAAGAACATCCTC GGCTGATGTAAGAAGGGATGA	60	76–100
18	MCW0330	17	TGGACCTCATCAGTCTGACAG AATGTTCTCATAGAGTTCCTGC	60	256–300
19	MCW0165	23	CAGACATGCATGCCCAGATGA GATCCAGTCCTGCAGGCTGC	60	114–118
20	MCW0069	26	GCACTCGAGAAAACTTCCTGCG ATTGCTTCAGCAAGCATGGGAGGA	60	158–176

**Table 3:** Number of alleles per locus per chicken breed

Locus	Breed						Overall

	BL-Nana	BR-Ff	BL-nana	BR-ff	BB	G	
MCW0020	3	6	6	4	3	3	8
LEI0166	6	6	6	5	6	4	8
MCW0111	4	4	5	4	5	5	7
MCW0034	4	3	3	4	3	4	5
MCW0067	3	6	5	4	4	6	8
MCW0165	7	5	7	7	8	5	12
MCW0020	4	4	4	5	5	3	8
MCW0103	6	4	5	5	5	4	8
MCW0165	4	5	5	4	4	3	5
MCW0104	8	6	8	7	8	7	13
MCW0069	6	6	7	4	4	4	8
MCW0014	5	4	6	5	6	4	7
ADL0278	6	5	7	6	5	3	10
MCW0248	6	5	6	6	8	6	9
LEI0234	9	5	7	7	7	5	15
MCW0123	3	5	5	3	3	2	10
MCW0081	6	5	3	3	4	4	6
MCW0183	6	5	5	6	6	5	9
MCW0295	5	6	4	4	4	5	7
ADL0268	3	5	5	5	5	5	9
Average	5.2±0.38	5.0±0.19	5.5±0.30	4.9±0.28	5.2±0.37	4.4±0.27	8.6±0.56

BL-Nana, heterozygous naked neck black; BR-Ff, heterozygous frizzled brown; BL-nana, normally feathered black; BR-ff, normally feathered brown; BB, black barred; G, gray.

Mean±standard error.

**Table 4:** Fixation indices (F_IT_, F_ST_, and F_IS_) and observed (H_O_) and expected (H_E_) heterozygosities per locus across six Saudi native chicken breeds

Locus	F_IT_	F_ST_	F_IS_	H_O_	H_E_	H_T_
MCW0020	0.00	0.04	−0.04	0.67	0.65	0.67
LEI0166	−0.09	0.03	−0.11	0.90	0.80	0.82
MCW0111	−0.24	−0.02	−0.22	0.77	0.62	0.61
MCW0034	−0.19	0.03	−0.22	0.78	0.64	0.64
MCW0067	0.06	0.01	0.05	0.66	0.71	0.71
MCW0165	0.03	0.02	0.01	0.80	0.83	0.85
MCW0020	−0.07	0.01	−0.09	0.72	0.68	0.69
MCW0103	−0.11	0.00	−0.11	0.87	0.77	0.77
MCW0165	0.01	0.04	−0.03	0.67	0.64	0.65
MCW0104	0.04	0.01	0.02	0.79	0.81	0.82
MCW0069	−0.12	−0.02	−0.11	0.71	0.66	0.65
MCW0014	−0.05	0.00	−0.05	0.83	0.77	0.77
ADL0278	−0.09	0.00	−0.09	0.71	0.65	0.66
MCW0248	−0.05	0.00	−0.05	0.83	0.82	0.81
LEI0234	0.01	0.05	−0.04	0.78	0.75	0.78
MCW0123	0.25	0.13	0.14	0.35	0.44	0.48
MCW0081	−0.23	0.02	−0.25	0.88	0.70	0.71
MCW0183	−0.14	0.04	−0.18	0.91	0.77	0.79
MCW0295	0.01	−0.01	0.03	0.74	0.75	0.74
ADL0268	−0.15	−0.01	−0.14	0.79	0.68	0.68
Overall	−0.06	0.02	−0.08	0.76	0.71	0.71

F_IT_, inbreeding coefficient of an individual relative to the total population; F_ST_, the effect of subpopulations compared with the total populations; F_IS_, inbreeding coefficient of an individual relative to the subpopulation;H_E_, average expected heterozygosity estimated from each population; H_T_, total gene diversity or expected heterozygosity in the total population as estimated from the pooled allele frequencies.

**Table 5:** Genetic variability estimates for 20 microsatellite loci in six native chicken populations

Breed	Fixation indices			Alleles/locus	Heterozygosity		dHWE
	
	F_IS_	F_IT_	F_ST_		H_O_	H_E_	
BL-Nana	−0.01	−0.07	0.02	5.20±1.70	0.72±0.15	0.68±0.12	2
BR-Ff	−0.09	−0.05	0.02	5.00±0.86	0.78±0.12	0.67±0.07	0
BL-nana	−0.08	−0.05	0.02	5.45±1.36	0.77±0.14	0.68±0.09	1
BR-ff	−0.10	−0.07	0.01	4.90±1.25	0.72±0.18	0.62±0.13	1
BB	−0.10	−0.05	0.02	5.15±1.63	0.78±0.16	0.67±0.13	0
G	−0.05	−0.06	0.02	4.35±1.23	0.77±0.27	0.67±0.14	0

BL-Nana, heterozygous naked neck black; BR-Ff, heterozygous frizzled brown; BL-nana, normally feathered black; BR-ff, normally feathered brown; BB, black barred; G, gray; H_O_, observed heterozygosity; H_E_, expected heterozygosity; dHWE,number of loci deviating from Hardy–Weinberg equilibrium after Bonferroni correction.

Mean±standard deviation.

**Table 6:** Genetic distance matrix among Saudi native chicken breeds

Breed	BL-Nana	BR-Ff	BL-nana	BR-ff	BB	G
BL-Nana	-					
BR-Ff	0.157	-				
BL-nana	0.107	0.122	-			
BR-ff	0.176	0.139	0.149	-		
BB	0.112	0.159	0.126	0.200	-	
G	0.159	0.210	0.168	0.246	0.234	-

BL-Nana, heterozygous naked neck black breed; BR-Ff, heterozygous frizzled brown breed; BL-nana, normally feathered black breed; BR-nana, normally feathered brown breed; BB, black-barred breed; G, gray breed.
